# Electrospun nanofibers of cellulose acetate/metal organic framework-third generation PAMAM dendrimer for the removal of methylene blue from aqueous media

**DOI:** 10.1038/s41598-023-32097-3

**Published:** 2023-03-25

**Authors:** Yasaman Heidari, Ebrahim Noroozian, Shahab Maghsoudi

**Affiliations:** Department of Chemistry, Shahid Bahonar University of Kerman, Kerman, Iran

**Keywords:** Analytical chemistry, Environmental chemistry

## Abstract

In this research, magnetic metal–organic framework nanofibers were produced by the electrospinning method. The nanocomposite was functionalized by third generation hyperbranched poly(amidoamine) dendrimer (PAMAM) to improve its dye adsorption efficiency from aqueous media. The characteristics of the synthesized magnetic nanocomposite was determined by Fourier-transform infrared spectroscopy (FTIR), X-ray diffraction (XRD), energy-dispersive X-ray spectroscopy (EDS) along with elemental mapping analysis and scanning electron microscopy (SEM). Central composite design (CCD) based on response surface methodology (RSM) was performed to optimize the adsorption variables and the values of coefficient of determination (R^2^) and adjusted R^2^ were 0.9837 and 0.9490, respectively. The results obtained demonstrated remarkable properties of the synthesized nanofiber as adsorbent for methylene blue from aqueous solutions with the removal efficiency of 95.37% and maximum methylene blue (MB) adsorption capacity of 940.76 mg g^−1^ under optimized conditions. In addition, it was shown that kinetics and adsorption isotherm of the dye removal process followed Langmuir and pseudo-second-order models, respectively. Thermodynamic study of the dye removal indicated that the process was spontaneous and favorable at higher temperatures. Also, the reusability study shows favorable dye removal efficiency of 80.67% even after 4 cycles. To investigate the performance of the adsorbent for the removal of MB in real samples, a sewage sample from a local hospital was used. The result showed good efficiency of the adsorbent.

## Introduction

Organic dyes are aromatic compounds that are mainly used in textile, leather, plastic, rubber, pharmaceutical and cosmetic industries. The presence of dyes in the wastewater of these industries is one of the most serious environmental concerns due to their possible adverse effects on human health. Textile industry wastewaters are a dominant source of environmental pollution. Because of the persistence of these dyes in the environment, their release without purification into aqueous media causes adverse effects on human health^1,2^. Methylene blue (MB) with chemical formula C_16_H_18_ClN_3_S, is one of the synthetic cationic dyes which are frequently used in biological processes in determining cell mortality and in textile industries for coloring silk, cotton and wool. The presence of MB in water (> 1 mg L^−1^) can affect human health and cause symptoms such as eye burns, itching, breathing difficulties and, vomiting^3–5^. Therefore, the process of wastewater treatment and pollution removal has become an important issue, recently. Until now, various physical and chemical processes have been utilized to combat dye pollution, such as filtration^6^, coagulation^7^, adsorption^8^, photocatalytic degradation^9^, etc.

Each purification method has its own advantages and disadvantages, and the choice of method mainly depends on the purpose of purification. For instance, coagulation is one of the most widely used processes for removing dyes from industrial wastewater. On the other hand, the use of coagulant leads to produce a large amount of chemical sludge which makes its disposal an important environmental issue. Membrane filtration processes show high removal efficiency, but with major problem of high cost of operation, which is not economically viable. Hence, due to its cost effectiveness, reliability, high efficiency and convenience, adsorption techniques have attracted attention for dye removal in recent years^10^. Up to now, various types of sorbent materials such as polymers^11^, activated carbon^12,13^ and zeolites^14^ have been studied for resolving this issue. In this respect, nonporous structures such as, metal–organic frameworks (MOFs) have frequently been used for pollution treatment, due to their unique structure, high specific surface area, easy functionalization and high porosity^15,16^. Because of its super chemical stability and excellent adsorption efficiency, zirconium-based MOF (UiO-66) has received significant attention and exhibits promising applications in various subjects, such as in pollutant removal, drug delivery, photo-catalysis, and fluorescence sensing^17–21^.

It has been shown that magnetite nanoparticles (Fe_3_O_4_) can be incorporated with metal–organic frameworks to improve adsorption efficiency and accelerate extraction and separation by using an external magnet^22^. Also, the surface of MOF can be functionalized to increase its adsorptive capacity. Up to now, various nanostructures have been studied, such as graphene oxide (GO)^23^, polymeric nanocomposite beads^24^, activated carbon^25^, bio-char composites^26^ and carbon nanotubes^27^. Among them, PAMAM-dendrimer, a hyperbranched polymer with internal cavities, a remarkable number of terminal functional groups, a highly cross-linked structure, and high adsorption capacity through increasing the generation, makes it a desirable candidate to be grafted onto the surface of MOFs for enhancing their dye adsorption capacity^28^. The tree-like structure and unique behavior of these macromolecules make them suitable in various applications. The crystalline solid powder MOF can be immobilized in a polymer solution as filler through an electrospinning process to produce MOF nanofibers with high specific surface area and chemical stability^29^. In electrospinning technique, the adsorbent polymer fibers are generated by stretching a drop of polymer solution to a counter electrode by applying an electric field^30^.

In this work, for the first time a core shell Fe_3_O_4_/MOF composite was synthesized by hydrothermal method and functionalized with the third generation, porous and multi-branched PAMAM dendrimer in order to increase its surface area and adsorption capacity. The modified polymer nanofibers produced by electrospinning technique were used as an adsorbent for the removal of MB from aqueous media. Six adsorptive parameters, including initial concentration of the MB dye, solution pH, adsorbent dosage, contact time, temperature, and mixing rate were optimized by applying central composite design (CCD) in response surface methodology (RSM). The isotherm, kinetics and thermodynamics of the dye removal were investigated.

## Experimental

### Materials

Ferric chloride hexahydrate (FeCl_3_·6H_2_O) and ferrous chloride tetrahydrate (FeCl_2_·4H_2_O) of ≥ 99% purity were obtained from Sigma Aldrich (Burlington, USA). Zirconium chloride (ZrCl_4_), methyl acrylate (CH_2_ = CHCOOCH_3_), terephthalic acid, TPA (C_6_H_4_(COOH)_2_), diethylenetriamine [(NH_2_CH_2_CH_2_)_2_NH), ethylenediamine (NH_2_CH_2_CH_2_NH_2_), cellulose acetate (CA), acetic acid, ammonia solution (25%), methanol, *N*,*N*-dimethylformamide (DMF), ethanol and MB were of analytical grade with assays of ≥ 99% and purchased from Merck (Darmstadt, Germany).

### Apparatus and software

A Tensor 27 FT-IR spectrometer from Brucker (Ettlingen, Germany) was employed to record the FT-IR spectra of the sorbent. A TESCAN-XMU scanning electron microscope (SEM) model MIRA3 (Brno, Czech) was used to determine the morphology of the sorbent surface. Thermaogravimetric analyzer (TGA/DTG) model STA 409 CD (EVISA, Germany) was utilized to measure thermal properties of the sorbent from 25 to 550 °C at a heating rate of 10 °C min^−1^. X-ray diffraction patterns (XRD) of the magnetite nanofiber was obtained by Malvern Panalytical-Spectris XRD analyzer (Egham, UK). A Trans Digital pH-meter model Bp3001 (Bukit Merah, Singapore) was used to adjusted the pH values. Design Expert software version 12.0.3.0 (Stat-Ease Inc., Minneapolis, USA) was employed for optimization of independent variables.

### Synthesis of PAMAM-functionalized Fe_3_O_4_@MOF

The PAMAM dendrimer (G3) was synthesized by repeating two types of reactions, i.e. Michael addition and amidation reactions, with slight modifications^31^. Briefly, the half generation (G0.5) was obtained by adding dropwise 25 mL of methanol solution of methyl acrylate into 5 mL of diethylenetriamine solution. Then, 40 mL of ethylenediamine was added and the mixture was stirred for 5 days at ambient temperature under an inert gas condition. Here, the first generation (G1) was obtained. To prepare generation (G1.5), 6.74 g of the (G1) dendrimer and 30 mL of methyl acrylate were mixed and kept stirring for 7 days at similar condition. Next, 60 mL ethylenediamine was added to the yellowish liquid and continued stirring for 10 days. Now, the second generation was obtained. In order to synthesize generation (G2.5), 62 mL methyl acrylate was added to (G2) and continued stirring for 15 days under N_2_ gas. The last generation dendrimer (G3) was prepared by adding 90 mL ethylenediamine to the previous generation and continued stirring for 21 days under the same condition (Supplementary Information 1).

**Figure 1:**
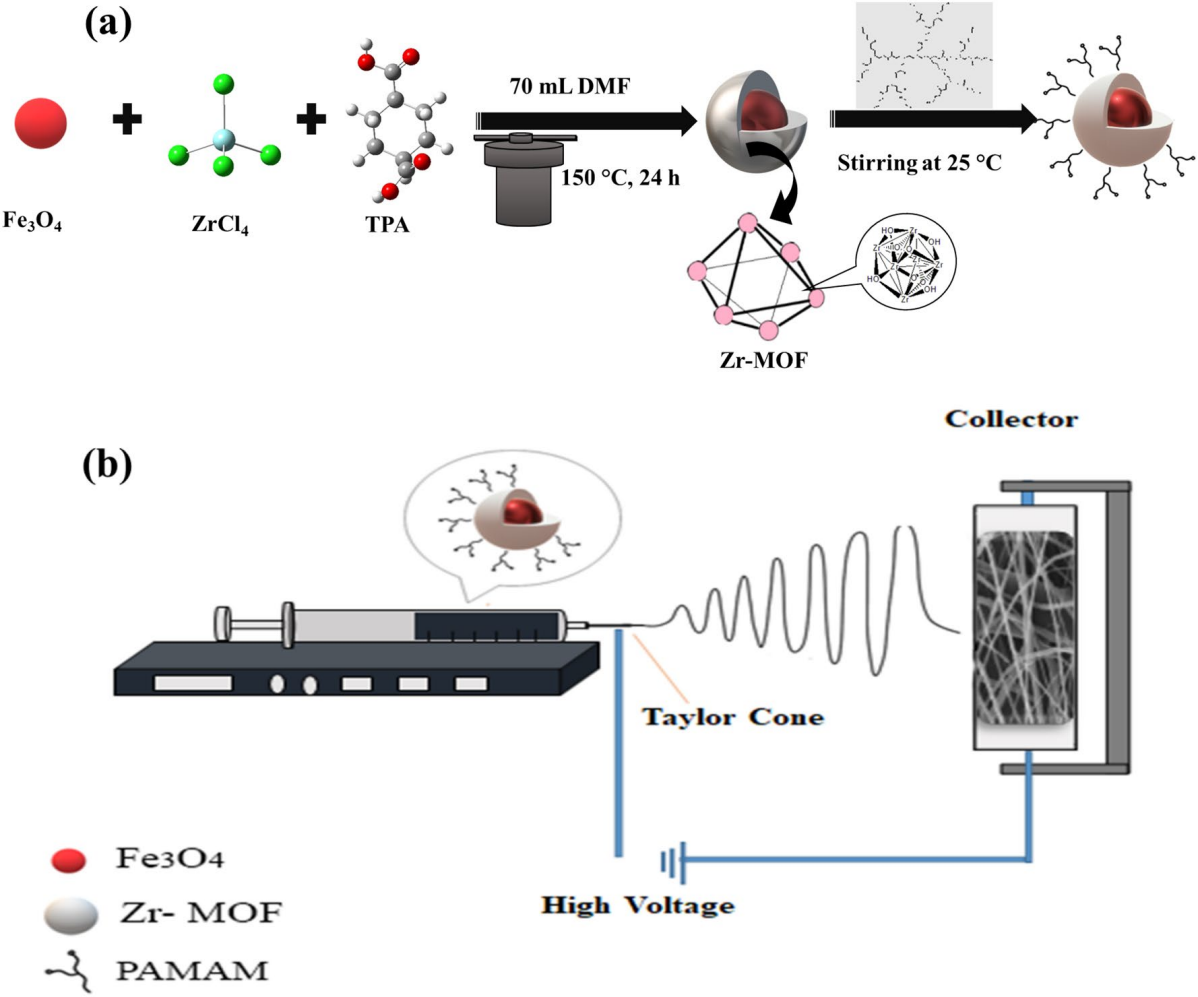
(**a**) The formation diagram of Fe_3_O_4_@MOF-PAMAM sorbent; (**b**) Schematic of the electrospinning apparatus.

Magnetite nanoparticles were prepared by the co-precipitation technique as reported by Chena with slight modifications^32^. Summarily, 4.6 g FeCl_3_·6H_2_O and 2.2 g FeCl_2_·4H_2_O were dissolved in 100 mL deionized water under N_2_ gas blanket and stirred. Then, 10 mL of 25% ammonia solution was added dropwise to the resulting solution in 30 min and continued mixing for 1 h at 60 °C. The magnetic nanoparticles produced were separated by a super magnet, and washed with deionized (DI) water and methanol and dried at ambient condition. For the preparation of the Fe_3_O_4_ @ MOF-PAMAM, 0.1 g of the prepared Fe_3_O_4_ nanoparticles were added to 70 mL a DMF solution containing ZrCl_4_ and terephthalic acid (molar ratio 1:1) under sonication for 30 min^22^. After adding 2 mL acetic acid, the suspension was heated in an oven at 150 °C for 24 h. The magnetic nanopowder obtained was separated with an external super magnet and washed with DI water and ethanol several times and dried in an oven at 60 °C for 24 h. The synthesized nanocomposite (0.2 g) was added to the prepared PAMAM methanol solution and homogenized by mechanical stirring at 25 °C. Then the sorbent was collected by the external magnet and allowed to be dried overnight after washing with methanol several times. The formation diagram of Fe_3_O_4_@MOF-PAMAM sorbent was shown in Fig. 1a.

### Electrospinning of cellulose acetate nanofibers

A CA polymer solution (25 w/v %) was prepared by adding 2.5 g CA into a mixture of DMSO/acetone (1:3) and stirred at ambient temperature. Next, various amounts of the synthesized Fe_3_O_4_@MOF-PAMAM nanocomposite (77, 101, 125, 166 mg) were added to the polymer solution and stirred. The homogenized solutions were loaded into 2.5 mL polyethylene syringe. The electrospining process was carried out under injection rate (1 mL h^−1^) and voltage 22 V with 12 cm distance between tailor cone and collector. After drying, the formed nanofibers were separated with the collector electrode. The Schematic of the electrospinning apparatus was depicted in Fig. 1b.

### Adsorption procedure

The Fe_3_O_4_@MOF-PAMAM sorbent was used to adsorb MB from aqueous solutions. The working dye solution of MB was prepared by diluting a stock solution (1000 mg L^−1^) in deionized water. The initial pH of the solution was adjusted by adding 0.1 M HCl or NaOH. Batch adsorption studies were carried out by addition of different amounts of sorbent (0.004–0.007 g L^−1^) to 10 mL aliquots of dye solution with initial concentration of (10–21 mg L^−1^) and stirred at (24–33 °C). During the removal process, the magnetic nanofibers were separated using an external magnet and the separated solution was analyzed by using UV–Vis spectrophotometry at maximum wavelength of 665 nm. The adsorption capacity (Q_t_) and the percentage removal (% R) at a given time (t), were calculated using Eqs. (1 and 2):

1 $${Q}_{t}=\frac{{C}_{0}-{C}_{t}}{M}V,$$

2 $$\% R=\frac{{(C}_{0}-{C}_{e})}{{C}_{0}}\times 100,$$

where C_0_ is the initial concentration of MB, C_t_ is its concentration at specific time t, C_e_ is the equilibrium concentration of MB (µg mL^−1^), M is the mass of the sorbent material used (g), V is the volume of the solution (L), and % R is the percentage of dye removed.

### Determination of zero point of charge of nanocomposite

Zero point of charge (ZPC) was determined as described elsewhere^2^. Briefly, 45 mL of KNO_3_ (0.1 M) solution was transferred to a series conical flask. Initial pH value was adjusted at 2–12 by addition of HCl and NaOH 0.1 M. Next, 0.1 g of nanocomposite was added to each flask and the suspensions were shaken manually and allowed to equilibrate for 48 h. The suspension was separated by the filtration and the final pH was recorded.

## Results and discussion

### Characterization

**Figure 2:**
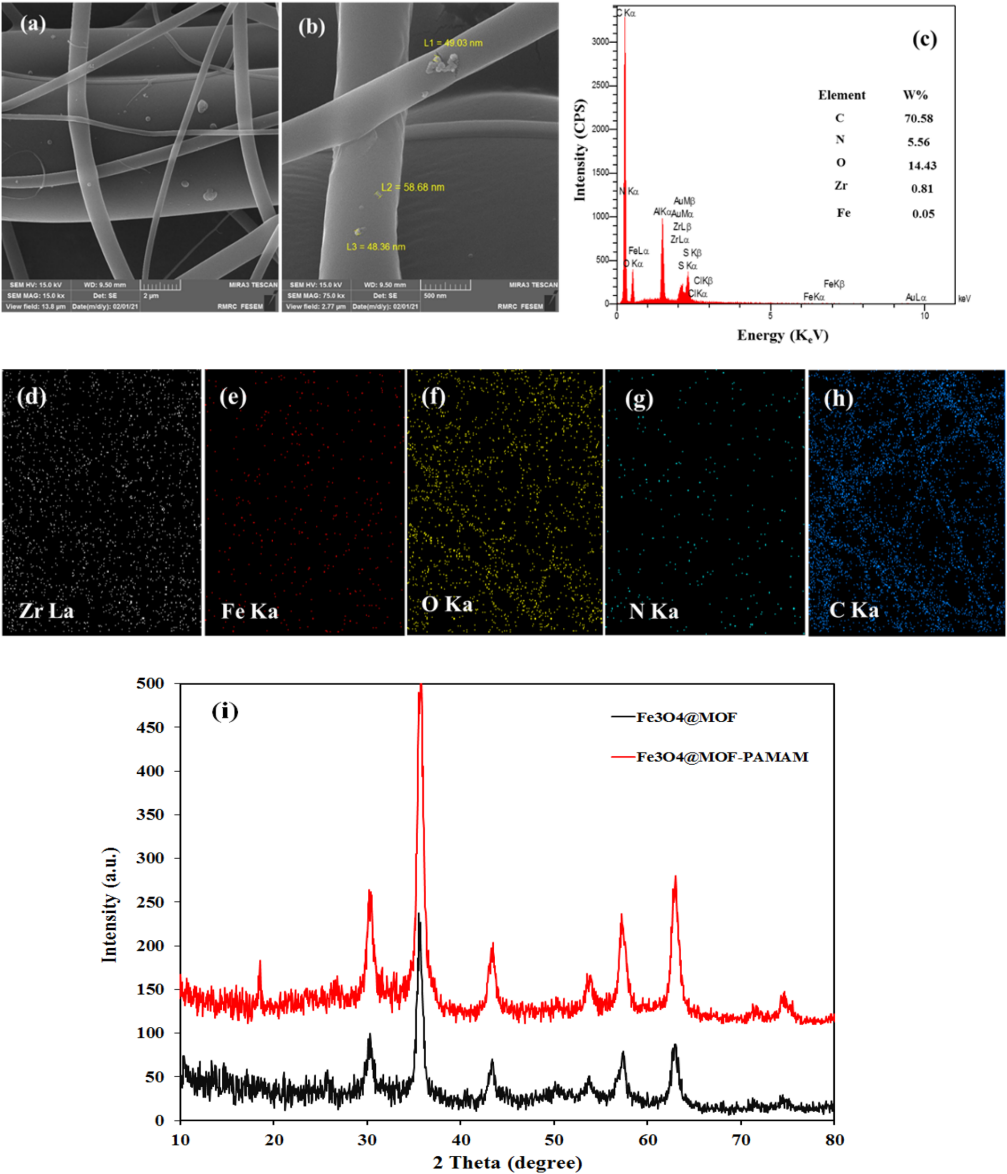
(**a**,**b**) FE-SEM images of the Fe_3_O_4_@MOF-PAMAM; (**c**) The EDS of Fe_3_O_4_@MOF-PAMAM; (**d**–**h**) The EDS-mapping of loaded Fe_3_O_4_@MOF-PAMAM; (**i**) XRD patterns of Fe_3_O_4_@MOF and Fe_3_O_4_@MOF-PAMAM.

The modification of the nanocomposite was characterized by FT-IR, FE-SEM, XRD, VSM, EDS and EDS-mapping. Figures 2a and 2b present the well-defined morphology with irregular microspheres shapes of PAMAM-functionalized Fe_3_O_4_@MOF with the average particle size of 40–50 nm distributed on the surface of the CA nanofibers. The EDS analysis of the magnetic nanofibers is shown in Fig. 2c. As can be seen, the peaks for Fe, C, Zr, O and N demonstrate the formation of adsorbent. In Fig. 2d–h, the image of EDS-mapping corroborated the homogeneous distribution of the magnetic sorbent on the CA nanofibers.

**Figure 3:**
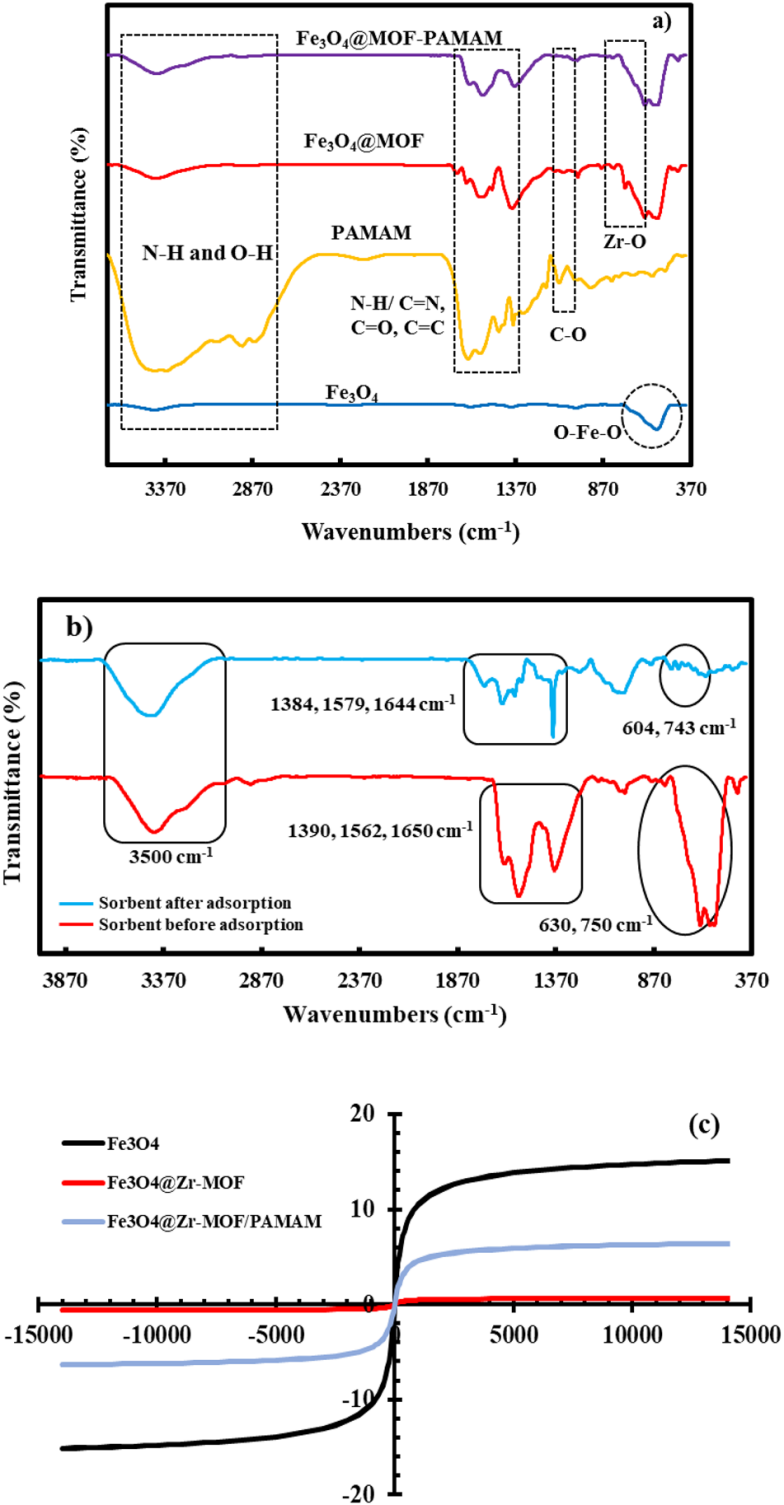
(**a**) FT-IR spectra of Fe_3_O_4,_ PAMAM, Fe_3_O_4_@MOF and Fe_3_O_4_@MOF-PAMAM; (**b**) FT-IR spectra of Fe_3_O_4_@MOF-PAMAM sorbent before and after adsorption of MB; (**c**) VSM hysteresis curves of the magnetic nanoparticles.

The XRD patterns of the Fe_3_O_4_@MOF and Fe_3_O_4_@MOF-PAMAM are depicted in Fig. 2i. The characteristic peaks at (2θ = 30, 35 and 53) are in agreement with the standard card (00-042-1164). No significance variation in the X-ray diffraction peaks of Zr-MOF and Zr-MOF-PAMAM are observed which confirms the stability of the crystal structure of MOF after modification with PAMAM. The diffraction peaks at (2θ = 35, 43 and 62) consistent with the standard card (01-075-1610) illustrates the cubic crystal structure of the magnetic nanoparticles. The above results prove that MOF and MOF- PAMAM were successfully produced on the surface of Fe_3_O_4_ by hydrothermal and polymerization processes.

The surface modified adsorbent was also studied by FT-IR (Fig. 3a). In the spectrum of Fe_3_O_4_ the absorption peaks at 555 and 3500 cm^−1^ are attributed to the O–Fe–O and O–H, respectively. The FT-IR sepctra of the Fe_3_O_4_@MOF and Fe_3_O_4_@MOF-PAMAM, two peaks at 630 and 750 cm^−1^ belong to Zr–O stretching vibration of the Zr-MOF. The absorption peaks at 1390, 1562 and 1650 cm^−1^ can be attributed to the carboxyl group, C=C and N–H/C=N stretching vibration of the ligand and PAMAM functional group, respectively. The peak at 3500 cm^−1^ belong to the N–H stretching vibration overlapped with O–H stretching of amine groups of dendrimer and adsorbed water, respectively. Furthermore, FT-IR spectra of the modified adsorbent was studied before and after MB removal process (Fig. 3b). As can be seen, similar peaks can be observed. It could be interpreted based on the presence of MB into pores of nanofibers. The vibration peaks of the nanocomposite adsorbed by the MB were reduced, due to the interaction between nanocomposite and MB molecules. On the other hand, electrostatic interactions causes the peak to shift. The proposed mechanism shows H-binding and electrostatic interaction between adsorbent and MB (Supplementary information 1). These results confirm that the physical adsorption of MB occurs on the surface of the nanocomposite.

Magnetic measurements of Fe_3_O_4_, magnetic MOF and Fe_3_O_4_@MOF-PAMAM nanocomposite were carried out by using VSM hysteresis in a magnetic field of − 15,000 to + 15,000 Oe (Fig. 3c). The magnetic saturation values found were (15), (0.6) and (6) emu g^−1^, respectively. The reduction in magnetization demonstrates the adequate coating of the surface of Fe_3_O_4_ nanoparticles. However, functionalization of Fe_3_O_4_@MOF by PAMAM dendrimer causes substantial increase of the saturation magnetization of the nanocomposite^22^.

**Figure 4:**
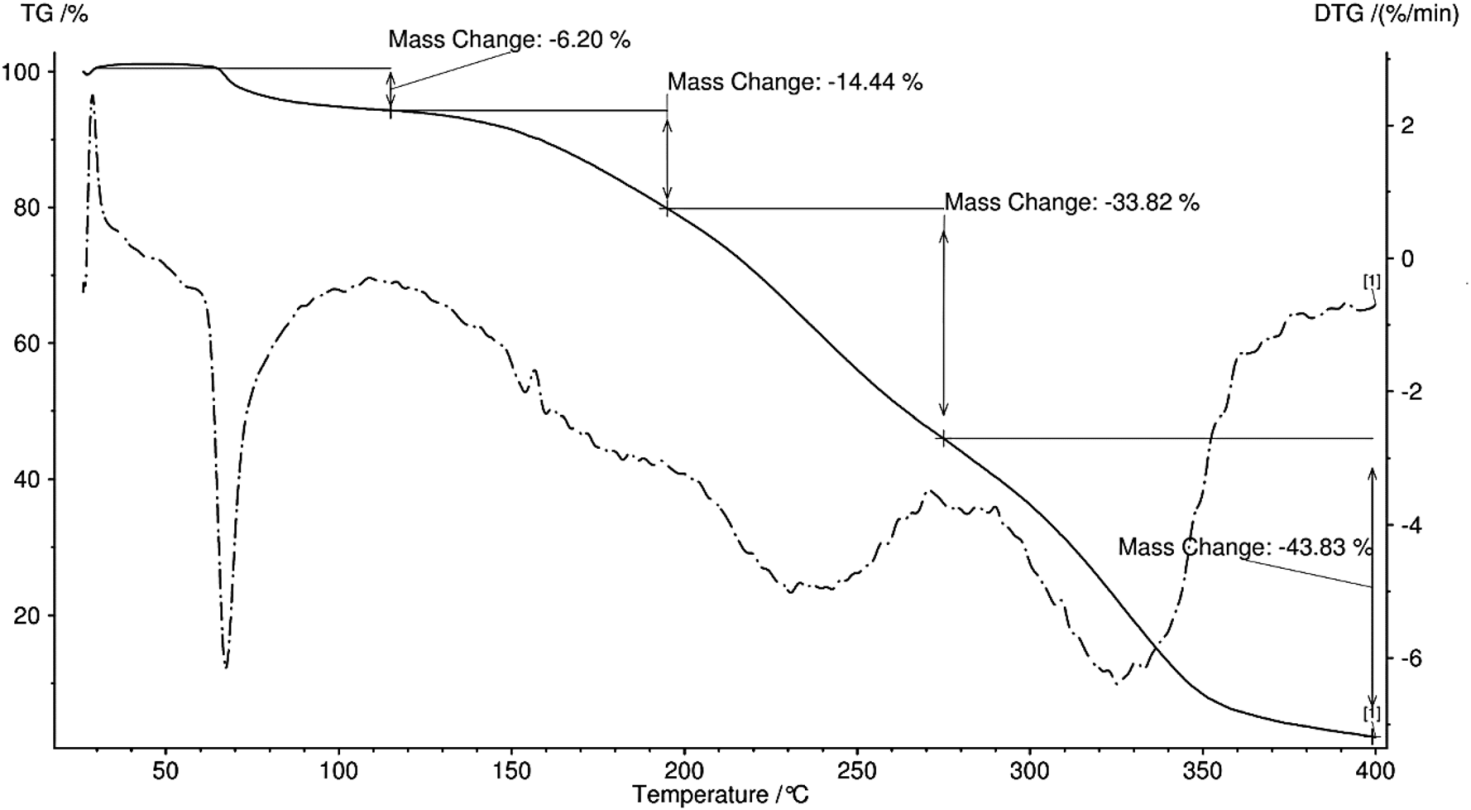
Thermogravimetric analysis of Fe_3_O_4_@MOF-PAMAM.

Thermogravimetric and derivative thermogravimetric curves of Fe_3_O_4_@MOF-PAMAM nanofibers are presented in Fig. 4. As observed, both curves show similar weight loss stages. The DTG curve exhibited three decomposition peaks at 67, 239 and 326 °C. The first slight weight loss of 6.2% at 67 °C, related to humidity of adsorbed and embedded water molecules into nanofibers. A significant thermal decomposition is seen between 200 and 350, which can be attributed to the degradation of the cellulose acetate chain and functional groups of the nanocomposite and complete decomposition of the nanofiber, which is consistent with the DTG curve results at 239 °C and 326 °C. This result showed that cellulose acetate nanofiber as a support layer allowed that the functional groups of nanocomposite remain available for the removal of MB from aqueous solution.

**Figure 5:**
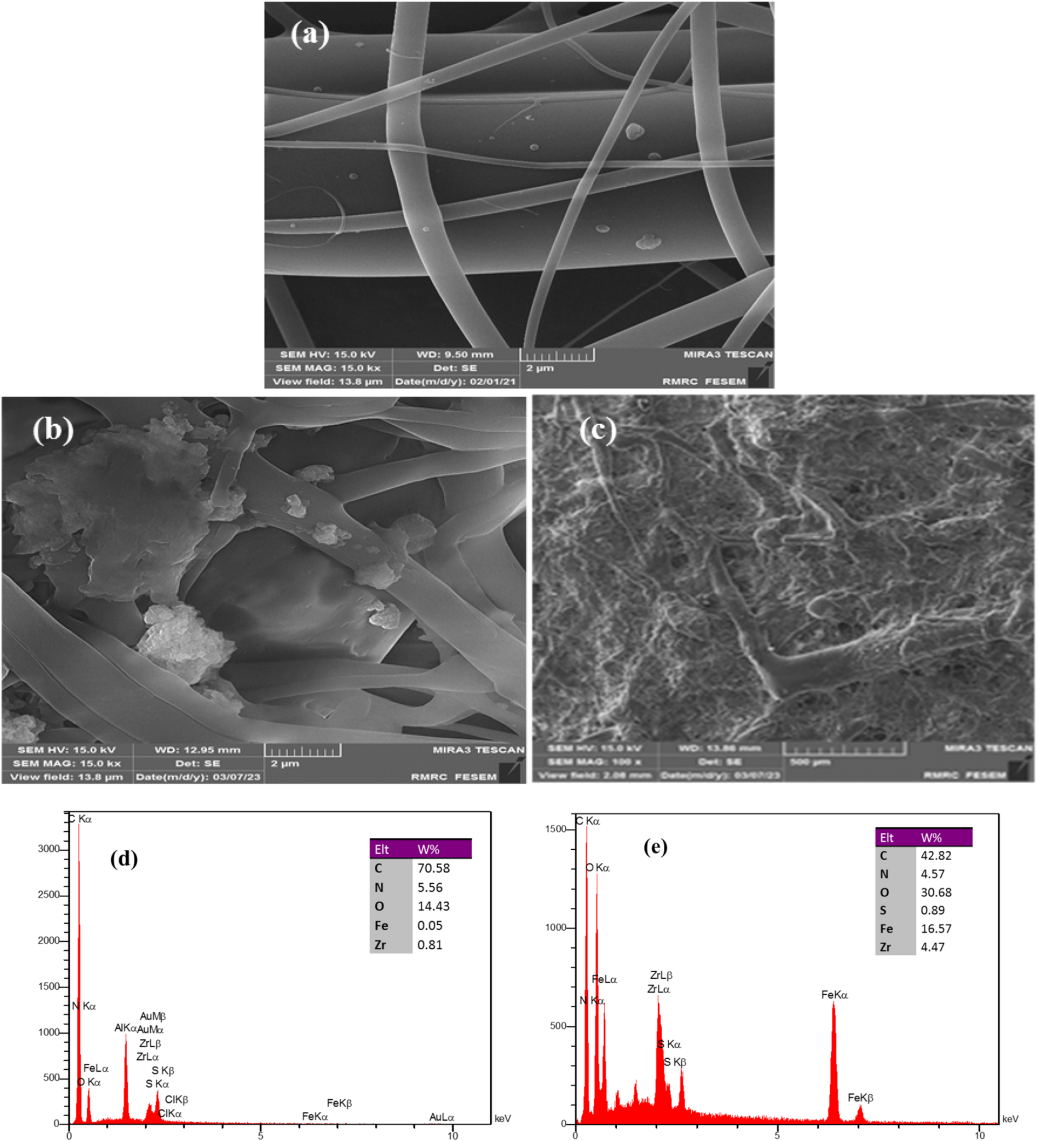
FE-SEM images of Fe_3_O_4_@MOF-PAMAM before (**a**) and after (**b**,**c**) adsorption of MB; EDX analysis of Fe_3_O_4_@MOF-PAMAM before (**d**) and after (**e**) adsorption of MB.

The change in the morphology of nanocomposite before and after MB adsorbed was investigated by FE-SEM analysis (Fig. 5a–c). The images illustrated the change in morphology caused by the absorption of MB molecules on the surface of PAMAM-functionalized Fe_3_O_4_@MOF nanofibers due to the porous and multi-branched structure of the nanocomposite. The results of EDS analysis shows presence of S element, in addition to the mentioned elements, which also confirm the absorption of MB molecules on the surface (Fig. 5d,e).

### Experimental design

Considering the inability of the one-at-a-time optimization method to provide interaction and curvature of variables, the response surface modeling (RSM) based on central composite design (CCD) was performed to design and optimize six practical variables of removal of the dye at five different levels. The parameters are listed in Table 1.

**Table 1:** Independent variables, their units and codes for the CCD design.

Parameters	Unit	Code	Levels				
			− α	− 1	0	+ 1	+ α
pH of solution (pH)	–	A	3	5.6	7.3	9	11.5
Adsorbent dosage (Dos)	g L^−1^	B	1.7	4	5.5	7	9.24
Time (t)	min	C	5.28	15	21.5	28	37.71
Initial concentration (C)	mg L^−1^	D	1.78	10	15.5	21	29.21
Temperature (T)	°C	E	17.27	24	28.5	33	39.72
Speed of stirring	rpm	F	40	70	90	110	140

To ensure orthogonality of the design, 50 treatments including 32 cubic, 10 axial and 6 center points were considered. After executing the CCD design matrix and receiving the responses, the RSM was established. Analysis of variance (ANOVA) was performed to evaluate the effect of the variables on the dye removal responses as presented in Table 2.

**Table 2:** Analysis of variance (ANOVA) for MB removal with Fe_3_O_4_@MOF-PAMAM imbedded in CA nanofibers.

Source	Sum of squares	df	Mean square	F-value	p-value	
Block	2749.35	2	1374.68			
Model	23,678.57	32	739.96	28.31	< 0.0001	Significant
A-pH	343.71	1	343.71	13.15	0.0025	
B-Dosage	1615.39	1	1615.39	61.80	< 0.0001	
C-Time	1458.87	1	1458.87	55.81	< 0.0001	
D-con	253.58	1	253.58	9.70	0.0071	
E-Tem	211.60	1	211.60	8.10	0.0123	
F-speed	3992.54	1	3992.54	152.74	< 0.0001	
AB	61.66	1	61.66	2.36	0.1454	
AC	1445.07	1	1445.07	55.28	< 0.0001	
AD	159.67	1	159.67	6.11	0.0259	
AE	112.50	1	112.50	4.30	0.0556	
BC	564.98	1	564.98	21.61	0.0003	
BD	115.75	1	115.75	4.43	0.0526	
BE	1537.07	1	1537.07	58.80	< 0.0001	
BF	136.54	1	136.54	5.22	0.0373	
CD	1367.65	1	1367.65	52.32	< 0.0001	
CE	323.09	1	323.09	12.36	0.0031	
CF	1549.02	1	1549.02	59.26	< 0.0001	
DE	524.88	1	524.88	20.08	0.0004	
DF	366.66	1	366.66	14.03	0.0019	
EF	93.98	1	93.98	3.60	0.0774	
A^2^	1562.40	1	1562.40	59.77	< 0.0001	
C^2^	620.59	1	620.59	23.74	0.0002	
D^2^	133.97	1	133.97	5.13	0.0388	
E^2^	1584.00	1	1584.00	60.60	< 0.0001	
ABE	180.88	1	180.88	6.92	0.0189	
BCE	262.89	1	262.89	10.06	0.0063	
BDE	187.02	1	187.02	7.15	0.0173	
BEF	515.85	1	515.85	19.73	0.0005	
CDE	101.03	1	101.03	3.87	0.0681	
DEF	1096.76	1	1096.76	41.96	< 0.0001	
A^2^B	599.29	1	599.29	22.93	0.0002	
A^2^D	582.03	1	582.03	22.27	0.0003	
Residual	392.09	15	26.14			
Lack of fit	191.43	12	15.95	0.2385	0.9698	Not significant
Pure error	200.66	3	66.89			
Cor total	26,820.01	49				

According to the results found, the term values less than the p-value of 0.05 imply a statistical effect on the dye removal. The relation between response (Y) and the coded factors was described by following equation:

3 $$\begin{aligned} {\text{Y}} & = { 66}.{48 } + {2}.{\text{78 A }} + {11}.{\text{39 B }} + {5}.{\text{73 C }} + {4}.{\text{51 D }} + {2}.{\text{18 E }} + {9}.{\text{48 F }} - {1}.{\text{39 AB }} + {6}.{\text{72 AC }} + {2}.{\text{23 AD }} \\ & \quad - {1}.{\text{87 AE }} - {4}.{2}0{\text{ BC }} - {1}.{9}0{\text{ BD }} + {6}.{\text{93 BE }} - {2}.0{\text{7 BF }} + {6}.{\text{54 CD }} + {3}.{\text{18 CE }} - {6}.{\text{96 CF }} + {4}.0{\text{5 DE }} \\ & \quad + {3}.{\text{38 DF }} + {1}.{\text{71 EF }} - {4}.{\text{83 A}}^{{2}} + {3}.0{\text{5 C}}^{{2}} + {1}.{\text{42 D}}^{{2}} + {4}.{\text{48 E}}^{{2}} - {2}.{\text{38 ABE }} + {2}.{\text{87 BCE }} - {2}.{\text{42 BDE }} \\ & \quad - {4}.0{\text{1 BEF }} - {1}.{\text{78 CDE }} + {17}.{\text{56 DEF }} - {8}.{\text{18 A}}^{{2}} {\text{B }} - {8}.0{\text{6 A}}^{{2}} {\text{D}}{.} \\ \end{aligned}$$

**Figure 6:**
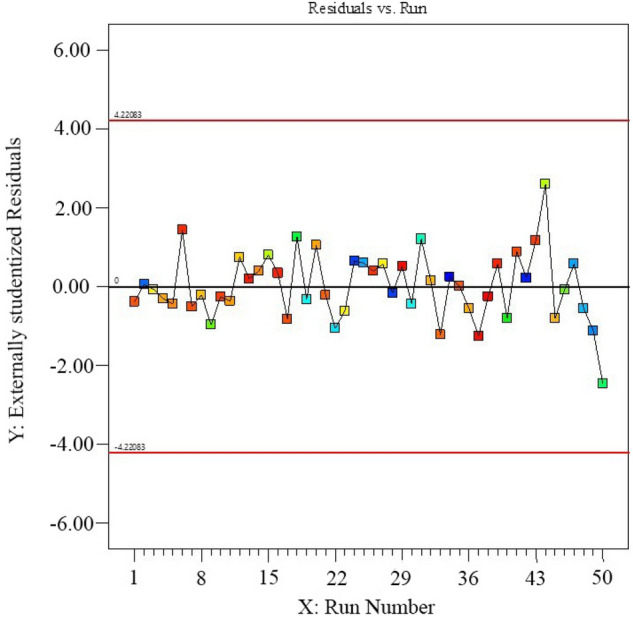
The plot of residual vs. runs for MB uptake capacity of the nanofibers adsorbent.

The statistical values of the Eq. (3) such as F-value (28.31), regression coefficient R^2^ (0.9837), adjusted R^2^ (0.9490), and predicted R^2^ (0.7756), confirmed that the model can describe the majority of response variation as a function of affecting parameters and their curvatures and interactions. In addition, the plots of residual vs. runs and random scatter in Fig. 6, are evidences that the proposed model can adequately be fitted on the experimental data. Evaluation of the RSM indicates that, several two and three factor interaction effects enroll in the model and it is very complicated. Among these factors, the AC, BC, BE, CD, CF, DE two factor interactions, and DEF three-factor interaction are most significant. These facts justified the importance of application of experimental design technique for obtaining real optimum coordinates in this work. The three-dimensional response surface plots (Supplementary Information 2) explain the impact of interaction between parameters on the adsorption efficiency. Consequently, the optimized coordinates (Table 3) of adsorption process was found by executing optimization process, implemented in design expert software, on the RSM. To test accuracy of RSM prediction, several experiments were carried out using the predicted optimum coordinates. As shown in Table 3, there is a good agreement between experimental and predicted responses.

**Table 3:** The predicted and experimental optimum responses.

pH	Dosage (g L^−1^)	Time (min)	Conc (mg L^−1^)	Temp (°C)	Speed (rpm)	Predicted response	Experimental response
8.4	5	28	21	24	70	96.67	93.82

### Zero point of charge (pH_ZPC_) of nanofibers

**Figure 7:**
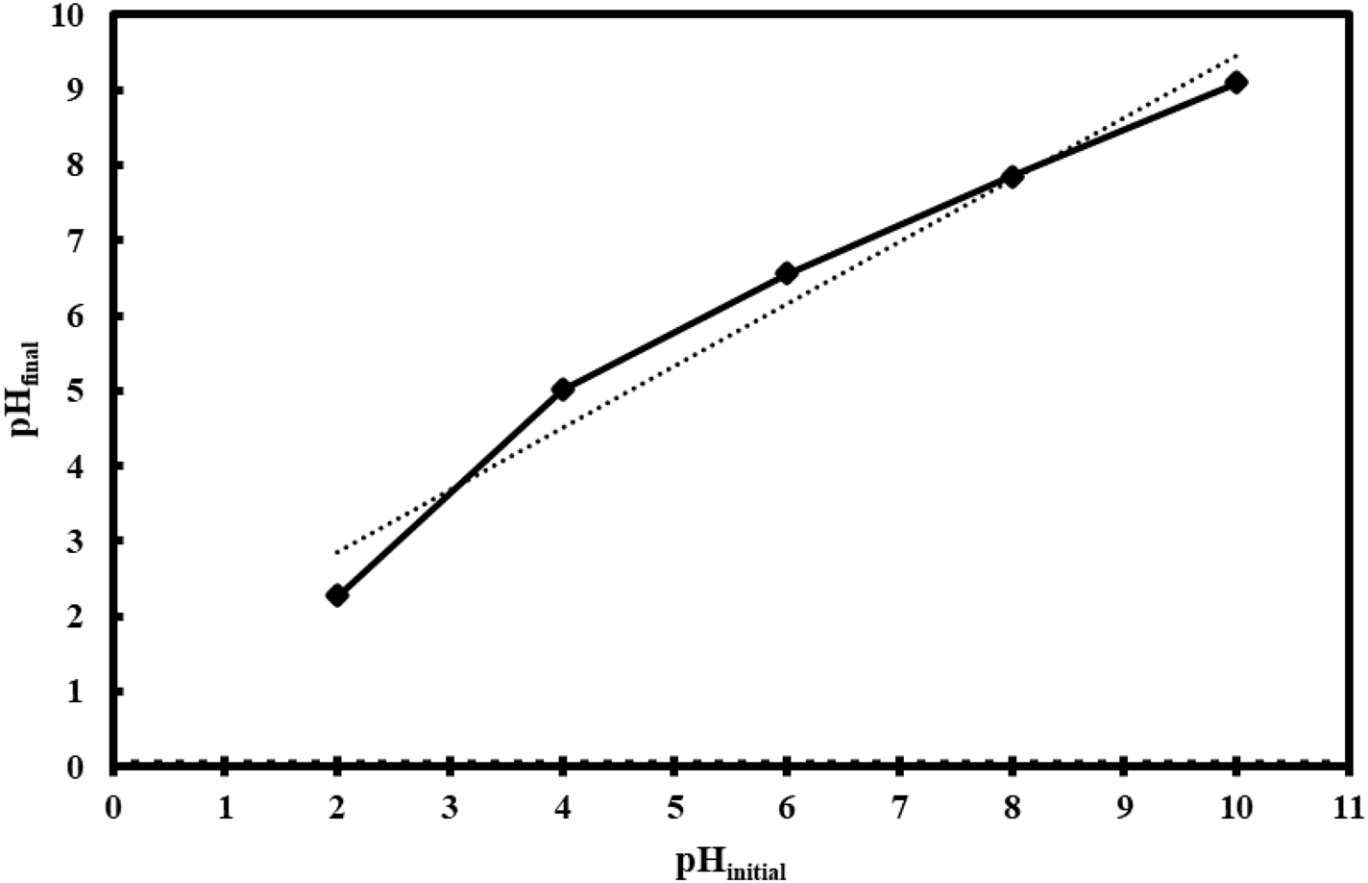
Zero point of charge of Fe_3_O_4_@MOF-PAMAM at various pH values.

The contradictory behavior of the sorbent in adsorption of MB can be interpreted by pH_ZPC_ value and molecular nature of MB. Hence, the results of the study of the zero point of charge is depicted in Fig. 7 by plotting the pH_final_ versus initial pH. At pH values lower than pH_ZPC_ (8), the surface of the sorbent is positive due to interaction of its functional groups with hydrogen ions of the medium, so rejection of cationic MB was increased. At alkaline condition (pH > pH_ZPC_), negative surface charge of the sorbent causes increasing adsorption of MB through electrostatic interaction.

### Reusability

**Figure 8:**
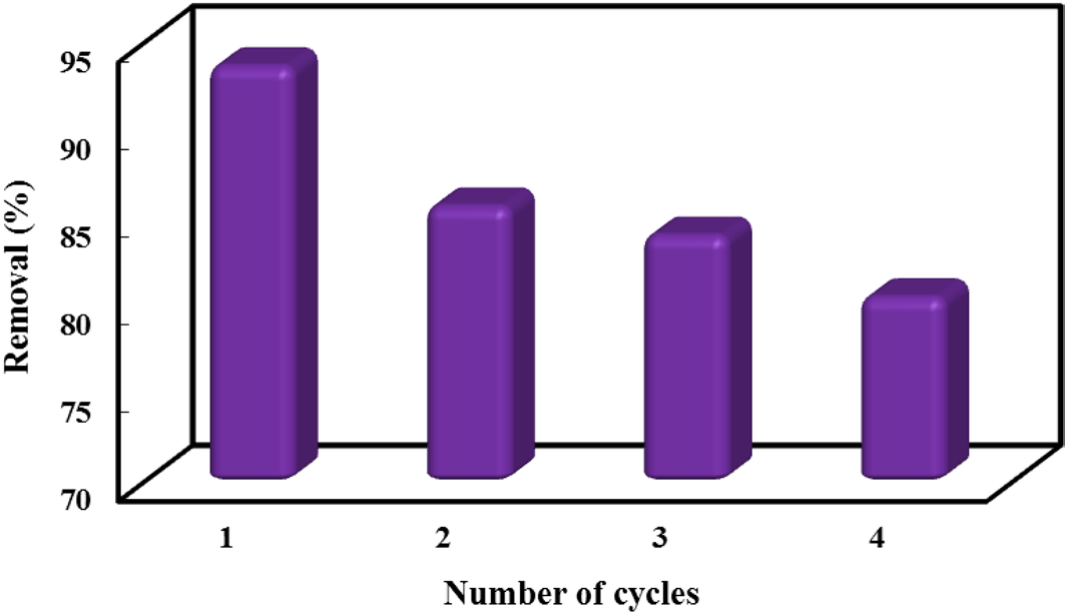
Reusability studies for the adsorption of MB dye onto Fe_3_O_4_@MOF-PAMAM.

The increase in industrial activities causes a large amount of sewage to be released into the environment and pollute it. As a result, it is very important to develop wastewater treatment processes without creating secondary waste. Therefore, regeneration is an environmentally friendly basic factor that determines practical application of the sorbent. For this purpose, after each adsorption operation, the loaded MB on the sorbent material was desorbed with ethanol under optimal condition and dried in an oven at 50 °C. The magnetic sorbent exhibited a decreasing removal efficiency after four cycles (Fig. 8 and Table 4). After the first cycle, the removal efficiency was slightly decreased by ~ 8% and reached up to 13% in cycle 4. This can be attributed to the fact that there are still enough empty active sites after the 4 cycle adsorption/desorption process. This result demonstrated that the magnetic nanosorbent could be a reusable adsorbent for the removal of MB from aqueous solutions.

**Table 4:** The results of reusability of the sorbent.

Cycles	Removal %	Adsorption capacity (mg/g)
1	93.82	39.40
2	85.85	36.05
3	84.18	35.35
4	80.67	33.88

### Adsorption properties

#### Adsorption kinetics

The mechanism of the adsorption kinetics on the sorbent was studied by the pseudo-first-order and pseudo-second-order models, as presented by Eqs. (4 and 5),

4 $$\mathrm{Log }\left({Q}_{e}-{Q}_{t}\right)=\mathrm{log}{Q}_{e}-\frac{{K}_{1}t}{2.303},$$

5 $$\frac{t}{q}=\frac{1}{{K}_{2}{q}_{e}^{2}}+\frac{t}{{q}_{t}},$$

where Q_t_ is the amount of adsorbed dye on the sorbent (mg g^−1^) at time t (min), and q_e_ is the amount of dye adsorbed on the sorbent at equilibrium (mg g^−1^), k_1_ is the rate constant of pseudo first-order (min^−1^) and k_2_ is the rate constant of pseudo second-order (g mg^−1^ min^−1^). The values of q_e_ and K_1_ can be obtained by plotting ln (q_e_–q_t_) versus t. Also, by plotting t/q_t_ vs t, the value of K_2_ and qe will be obtained from the intercept and slope of the line, respectively.

**Figure 9:**
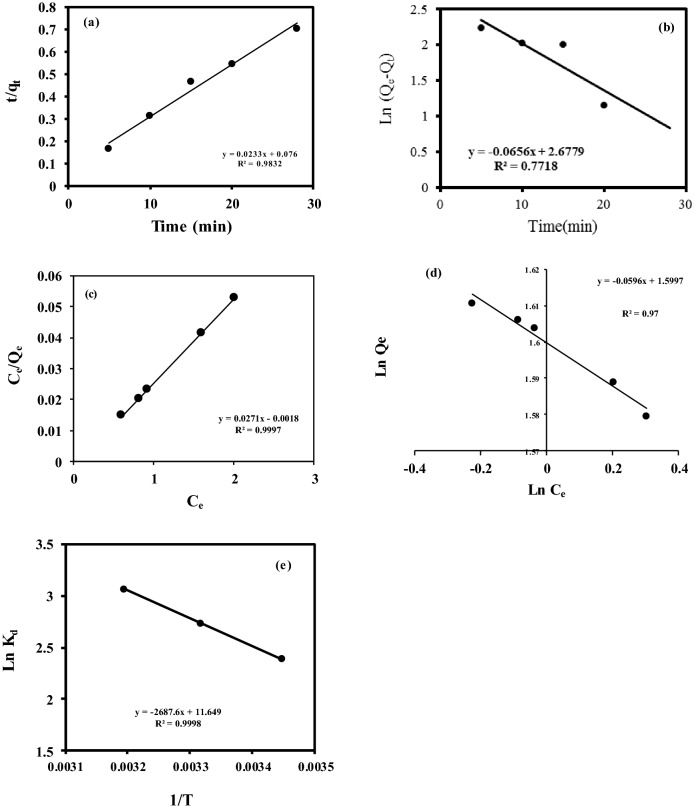
(**a**) Pseudo second-order; (**b**) Pseudo-first-order Lagergren adsorption curves; (**c**) Langmuir; (**d**) Freundlich adsorption isotherms; (**e**) Thermodynamics of adsorption of MB by the synthesized nanofibers (Temp: 24 °C, Dose: 0.005 g L^−1^, Concentration: 21 mg L^−1^, Time: 5–28 min, pH: 8.4, Stirring speed: 70 rpm).

The results presented in Table 3, show that the MB removal corresponds to the pseudo-second-order model. The curves of pseudo-first-order and pseudo-second-order models are exhibited in Fig. 9a,b.

#### Adsorption isotherm

Generally, two Langmuir and Freundlich models are perused to evaluate the appropriation mechanism of adsorption. The Langmuir mechanism is expressed as a single layer adsorption at constant temperature. The obtained data are measured by fitting to the following equation:

6 $$\frac{Ce}{Qe} =\frac{1}{{K}_{l}} + \frac{{C}_{e}}{{Q}_{m}},$$

where Q_e_ is the amount of solute adsorbed on the surface at equilibrium (mg g^−1^), C_e_ is the equilibrium concentration of adsorbate (mg L^−1^), K_l_ is Langmuir constant (mg L^−1^) and Q_m_ is the maximum adsorption capacity (mg g^−1^). By drawing C_e_/Q_e_ versus C_e_, the value of K_l_ and Q_m_ are calculated.

Contrary to Langmuir, Freundlich model is based on multiple layer adsorption with asymmetric distribution over the surface. The Freundlich isotherm is expressed by the following equation:

7 $${q}_{e=}{K}_{f}{C}_{e}^{1/n},$$

where 1/n is the adsorption intensity and K_f_ is the adsorption capacity constant (mg g^−1^). The value of K_f_ (slope) and n (intercept) can be obtained by plotting log (q_e_) against log (Ce). By comparing the R^2^ values of the two models, it is found that the Langmuir models (0.9997) is a better fit to the isotherm and therefore a single layer adsorption process is followed. The evaluated adsorption isotherm parameters are listed in Table 5 and the plots of Langmuir and Freundlich models are presented in Fig. 9c,d.

**Table 5:** Kinetic and isotherm parameters for the removal of MB.

Pseudo-second-order			Pseudo-first-order		
R^2^	q_e_	K_2_	R^2^	q_e_	K_1_
0.98	42.91	0.30	0.771	0.002	− 0.15

Freundlich constant			Langmuir constant		
R^2^	n	K_f_	R^2^	q_m_	K_1_
0.9760	− 16.77	0.0251	0.9997	36.90	15.17

#### Adsorption thermodynamics

Energy conversion and transfers always occur during the adsorption process, and due to that, the enthalpy, entropy and free energy parameters of the system change, which can be explained using thermodynamics parameters. Based on the thermodynamic parameter of enthalpy, the endothermic or exothermic absorption process is determined. Also, the irregularity of the absorption system and the spontaneity of the absorption reaction are determined using the entropy parameter and Gibbs free energy, respectively. The thermodynamic parameters of Gibbs free energy (∆G^o^), enthalpy change (∆H^o^) and entropy change (∆S^o^), are measured to evaluate the temperature dependency of adsorption process.

These parameters are determined by using the following equations:

8 $$\mathrm{\Delta G }=\mathrm{ \Delta H }-\mathrm{ T\Delta S},$$

9 $$\mathrm{ln}{K}_{d }= \left(\frac{\mathrm{\Delta H}}{\mathrm{RT}}\right)+ \left(\frac{\mathrm{\Delta S}}{\mathrm{R}}\right).$$

The plot of ln K_d_ vs. 1/T for the present work is shown in Fig. 9e. The values of ΔS° and ΔH° were obtained from the intercept and slope of the line, respectively. The obtained data are summarized in Table 6. The positive value of ΔH° indicates that the dye removal is an endothermic process. In endothermic adsorption processes, the adsorption capacity of the adsorbent increases with increasing temperature, and in fact, temperature is the controlling parameter of the adsorption interactions between the adsorbent and the adsorption molecules^33^. The negative value of ΔG° indicates that the adsorption process is spontaneous and the rise of the absolute value of ΔG° with increasing temperature indicates the spontaneous nature of the absorption process. In addition, it shows that the activation energy of the absorption process increases with the increase in temperature^34^.

**Table 6:** Parameters of thermodynamic for MB adsorption.

Thermodynamic parameters	ΔH (kJ mol^−1^)	ΔS (kJ Kmol^−1^)	ΔG (kJ mol^−1^)		
			290 K	301 K	313 K
Fe_3_O_4_@MOF/PAMAM	48.3874	0.1796	− 3.6966	− 5.762	− 7.8274

### Real samples

The efficiency of proposed sorbent for the analysis of real wastewater was investigated. A biological sample from the sewage of a local hospital was used as the real sample. The adsorption process of MB in the sewage sample was evaluated at 665 nm^−1^. The removal percentage of the MB from the wastewater reached more than 87% under optimized condition.

### Comparison with other methods

Comparison of the synthesized magnetic nanofiber with other sorbents for the adsorption of MB is shown in Table 7. Considering the results obtained in this work, it seems that the present magnetic sorbent has a favorable removal efficiency in comparison to other sorbents. The high dye removal efficiency with the lowest amount of Fe_3_O_4_@MOF-PAMAM, indicates the excellent potential of the present adsorbent for the removal of MB and other wastewater pollutants.

**Table 7:** Comparison of adsorption of MB with other methods.

Adsorbent	Optimal parameters				% R	Adsorption capacity (mg/g)	References
	pH	t (min)	C_0_ (mg L^−1^)	m (g L^−1^)			
Polyamide-vermiculite	5	60	15	0.15	99	76.42	^35^
Graphene oxide-persimmon tannin	8	180	35	0.02	92	256.58	^36^
Boron carbide (B4C)	7	90	10	0.1	97.3	188.68	^4^
Fava bean peels, *Vicia faba* (FBP)	5.8	40	50	5	90	140	^37^
Algerian kaolin	9	120	100	1	98	52.76	^38^
Fe_3_O_4_@MOF-PAMAM	8.4	28	21	0.005	95.37	940.76	This work

## Conclusion

In this study, the Fe_3_O_4_@MOF was functionalized with hyperbranched third generation PAMAM dendrimer (G3) and successfully applied for the removal of MB from aqueous media after incorporating it into cellulose acetate nanofibers by an electrospining technique. Due to certain limitations of commonly used MOF adsorbents for wastewater treatment, such as the low capacity of adsorption, coating with PAMAM can significantly improve their applicability in wastewater treatment. This is due to the presence of numerous amine terminal groups which enhance reactivity and solubility of the dendrimer. Therefore, the present PAMAM dendrimer can be the adsorbent of choice for the removal of MB. Additionally, combination of MOF with Fe_3_O_4_ enhanced the chemical stability of the sorbent and performance of adsorption. The functionalization process introduced many functional groups and active sites on the surface of the sorbent. Indeed, high porosity of MOF and introduction of PAMAM functional group at the surface of the sorbent play significant roles in improving the adsorption efficiency of the sorbent. The characterization of the sorbent by FTIR, TGA, XRD, EDS along with elemental mapping analysis and SEM demonstrated successful growth of MOF-PAMAM on the surface of Fe_3_O_4_ by hydrothermal and polymerization processes. Optimal experimental parameters consisting of initial concentration of MB (21 mg L^−1^), sorbent dosage (0.005 g L^−1^), contact time (28 min), pH (8.4), speed of stirring (70 rpm) and temperature (297 K) were found by CCD experimental design combined with RSM and the value of coefficient of variation (R^2^ = 0.9837) indicated a good correlation between the independent parameters and results. This magnetic nanofiber sorbent could be reused by desorption of the dye from the adsorbent. The adsorption performance of the nanofiber fits the pseudo-second-order kinetic and Langmuir isotherm models. The thermodynamic parameters, ΔG° and ΔH°, with respectively negative and positive values exhibited the removal process is spontaneous and endothermic and the reusability study indicated that the absorbent can be used as a renewable absorbent at least four cycles. The suitability of this adsorbent was evaluated by analyzing a real wastewater sample. The results indicate that present magnetic nanofibers could be a good sorbent for the removal of MB from wastewaters samples. Considering the results obtained, the application of the present magnetic nanofiber can be expanded to other environmental treatments.

## Supplementary Information

Supplementary Information 1.

Supplementary Information 2.

## Data Availability

The datasets used and/or analyzed during the current study are available from the corresponding author on reasonable request.

## References

[1] Isik B, Ugraskan V, Cankurtaran O (2022). Effective biosorption of methylene blue dye from aqueous solution using wild macrofungus (*Lactarius piperatus*). Sep. Sci. Technol..

[2] Murugesan A, Divakaran M, Raveendran P, Nitin Nikamanth A, Thelly KJ (2019). An eco-friendly porous poly (imide-ether) s for the efficient removal of methylene blue: Adsorption kinetics, isotherm, thermodynamics and reuse performances. J. Polym. Environ..

[3] Chowdhury S, Saha PD (2012). Biosorption of methylene blue from aqueous solutions by a waste biomaterial: Hen feathers. Appl. Water Sci..

[4] Ugraskan V, Isik B, Yazici O (2022). Adsorptive removal of methylene blue from aqueous solutions by porous boron carbide: Isotherm, kinetic and thermodynamic studies. Chem. Eng. Commun..

[5] Fito J, Abrham S, Angassa K (2020). Adsorption of methylene blue from textile industrial wastewater onto activated carbon of *Parthenium hysterophorus*. Int. J. Environ. Res..

[6] Kamari S, Shahbazi A (2020). Biocompatible Fe_3_O_4_@ SiO_2_-NH_2_ nanocomposite as a green nanofiller embedded in PES-nanofiltration membrane matrix for salts, heavy metal ion and dye removal: Long-term operation and reusability tests. Chemosphere.

[7] Dalvand A (2017). Application of chemical coagulation process for direct dye removal from textile wastewater. J. Environ. Health. Sustain. Dev..

[8] Achieng GO, Kowenje CO, Lalah JO, Ojwach SO (2021). Synthesis and characterization of FSB@ Fe_3_O_4_ composites and application in removal of indigo carmine dye from industrial wastewaters. Environ. Sci. Pollut. Res..

[9] Vuggili SB, Kadiya K, Gaur UK, Sharma M (2021). Synthesis of graphitic carbon nitride/cadmium sulfide core-shell nanofibers for enhanced photocatalysis. Environ. Sci. Pollut. Res..

[10] Pei R (2020). 3D-Printed metal-organic frameworks within biocompatible polymers as excellent adsorbents for organic dyes removal. J. Hazard. Mater..

[11] Lu Y (2020). A microporous polymer ultrathin membrane for the highly efficient removal of dyes from acidic saline solutions. J. Membr. Sci..

[12] Moosavi S (2020). Application of efficient magnetic particles and activated carbon for dye removal from wastewater. ACS Omega.

[13] Tang X, Ran G, Li J, Zhang Z, Xiang C (2021). Extremely efficient and rapidly adsorb methylene blue using porous adsorbent prepared from waste paper: Kinetics and equilibrium studies. J. Hazard. Mater..

[14] Mittal H, Babu R, Dabbawala AA, Stephen S, Alhassan SM (2020). Zeolite-Y incorporated karaya gum hydrogel composites for highly effective removal of cationic dyes. Colloids Surf. A Physicochem. Eng. Asp..

[15] Guo C (2020). Zeolitic imidazolate framework cores decorated with Pd nanoparticles and coated further with metal–organic framework shells (ZIF-8@ Pd@ MOF-74) as nanocatalysts for chemoselective hydrogenation reactions. ACS Appl. Nano Mater..

[16] Huang L, He M, Chen B, Hu B (2018). Magnetic Zr-MOFs nanocomposites for rapid removal of heavy metal ions and dyes from water. Chemosphere.

[17] Chen C (2017). Adsorption behaviors of organic micropollutants on zirconium metal–organic framework UiO-66: Analysis of surface interactions. ACS Appl. Mater. Interfaces..

[18] Sun Y (2020). Adsorptive removal of dye and antibiotic from water with functionalized zirconium-based metal organic framework and graphene oxide composite nanomaterial Uio-66-(OH) 2/GO. Appl. Surf. Sci..

[19] Jarai BM (2020). Evaluating UiO-66 metal–organic framework nanoparticles as acid-sensitive carriers for pulmonary drug delivery applications. ACS Appl. Mater. Interfaces..

[20] Chen X (2020). NH2-UiO-66 (Zr) with fast electron transfer routes for breaking down nitric oxide via photocatalysis. Appl. Catal. B: Environ..

[21] Yang X-L (2021). Selective dual detection of H2S and Cu2+ by a post-modified MOF sensor following a tandem process. J. Hazard. Mater..

[22] Far HS (2020). PPI-dendrimer-functionalized magnetic metal–organic framework (Fe_3_O_4_@ MOF@ PPI) with high adsorption capacity for sustainable wastewater treatment. ACS Appl. Mater. Interfaces..

[23] Firouzjaei MD, Afkhami FA, Esfahani MR, Turner CH, Nejati S (2020). Experimental and molecular dynamics study on dye removal from water by a graphene oxide-copper-metal organic framework nanocomposite. J. Water Process. Eng..

[24] Abdi J, Abedini H (2020). MOF-based polymeric nanocomposite beads as an efficient adsorbent for wastewater treatment in batch and continuous systems: Modelling and experiment. Chem. Eng. J..

[25] Wang H (2021). Effective adsorption of dyes on an activated carbon prepared from carboxymethyl cellulose: Experiments, characterization and advanced modelling. Chem. Eng. J..

[26] Navarathna CM (2020). Rhodamine B adsorptive removal and photocatalytic degradation on MIL-53-Fe MOF/magnetic magnetite/biochar composites. J. Inorg. Organomet. Polym. Mater..

[27] Sang X (2020). Interfacial growth of metal–organic framework on carboxyl-functionalized carbon nanotubes for efficient dye adsorption and separation. Anal. Methods..

[28] Wang Y, Wang J, Gao M, Zhang X (2017). Functional dual hydrophilic dendrimer-modified metal-organic framework for the selective enrichment of N-glycopeptides. Proteomics.

[29] Efome JE, Rana D, Matsuura T, Lan CQ (2018). Insight studies on metal-organic framework nanofibrous membrane adsorption and activation for heavy metal ions removal from aqueous solution. ACS Appl. Mater. Interfaces..

[30] Xue J, Wu T, Dai Y, Xia Y (2019). Electrospinning and electrospun nanofibers: Methods, materials, and applications. Chem. Rev..

[31] Alinezhad H, Amiri A, Tarahomi M, Maleki B (2018). Magnetic solid-phase extraction of non-steroidal anti-inflammatory drugs from environmental water samples using polyamidoamine dendrimer functionalized with magnetite nanoparticles as a sorbent. Talanta.

[32] Chen L, Xu Z, Dai H, Zhang S (2010). Facile synthesis and magnetic properties of monodisperse Fe_3_O_4_/silica nanocomposite microspheres with embedded structures via a direct solution-based route. J. Alloys. Compd..

[33] Al-Ghouti MA, Al-Absi RS (2020). Mechanistic understanding of the adsorption and thermodynamic aspects of cationic methylene blue dye onto cellulosic olive stones biomass from wastewater. Sci. Rep..

[34] Murugesan A, Mahendran P (2020). High-performance polyimides with pendant fluorenylidene groups: Synthesis, characterization and adsorption behaviour. J. Polym. Environ..

[35] Basaleh AA, Al-Malack MH, Saleh TA (2019). Methylene Blue removal using polyamide-vermiculite nanocomposites: Kinetics, equilibrium and thermodynamic study. J. Environ. Chem. Eng..

[36] Wang Z, Gao M, Li X, Niang J, Zhou Z, Li G (2020). Efficient adsorption of methylene blue from aqueous solution by graphene oxide modified persimmon tannins. Mater. Sci. Eng..

[37] Bayomie OS, Kandeel H, Shoeib T, Yang H, Youssef N, El-Sayed MM (2020). Novel approach for effective removal of methylene blue dye from water using fava bean peel waste. Sci. Rep..

[38] Mouni L (2018). Removal of methylene Blue from aqueous solutions by adsorption on Kaolin: Kinetic and equilibrium studies. Appl. Clay. Sci..

