# Supplementary material for: Electrospun nanofibers of cellulose acetate/metal organic framework-third generation PAMAM dendrimer for the removal of methylene blue from aqueous media
Source: Sci Rep. 2023 Mar 25;13:4924. doi: 10.1038/s41598-023-32097-3 (PMC10039946; doi:10.1038/s41598-023-32097-3)
Supplement: Supplementary file 1 — Supplementary Information 1. [file 41598_2023_32097_MOESM1_ESM.doc]

**Supplementary information 1**

(a)

(b)


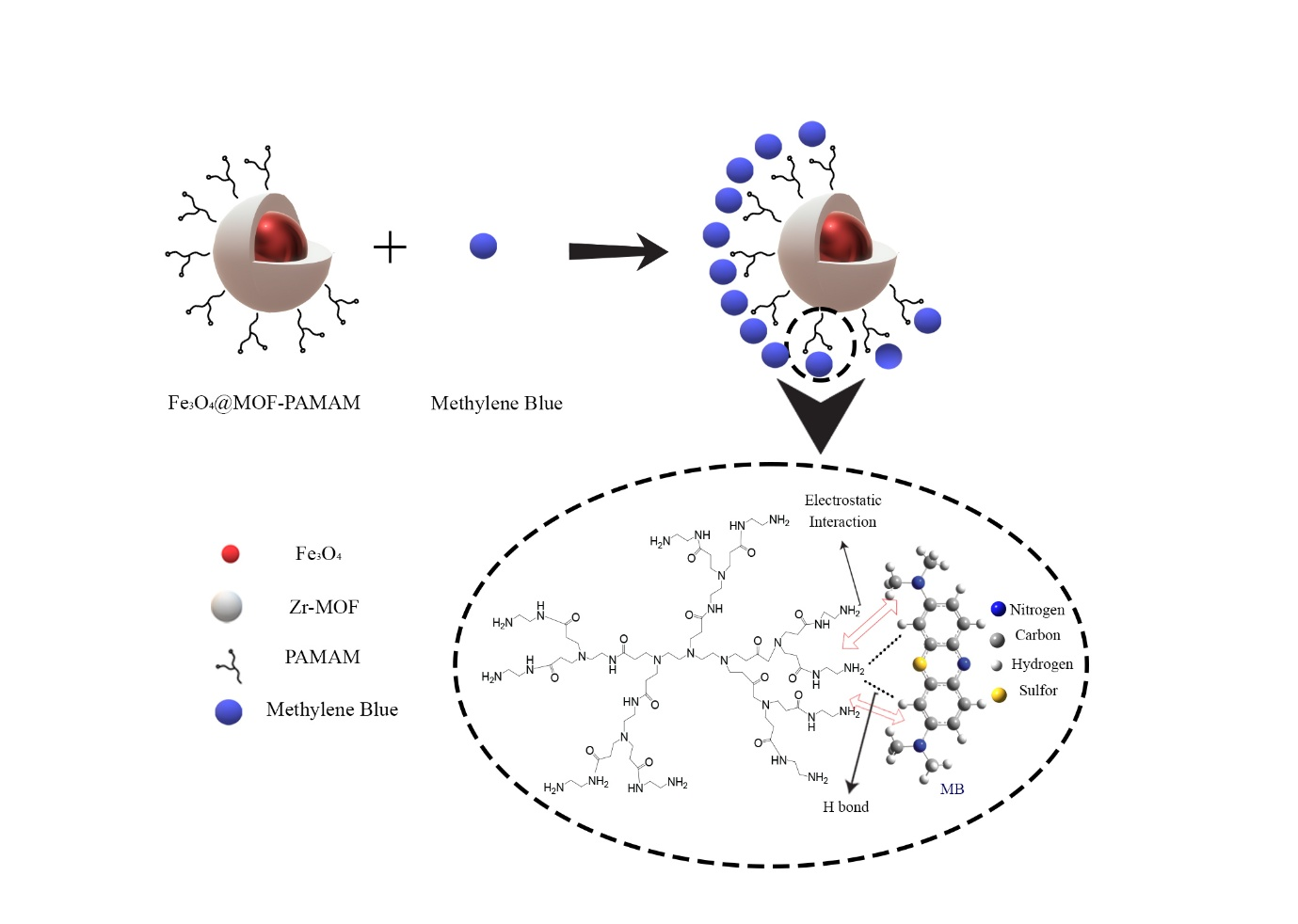


**Supplementary information 1:** Procedures for the preparation of PAMAM dendrimer (G3) (a), Possible mechanism of adsorption MB on the nanofiber sorbent (b).
