# Supplementary material for: Electrospun nanofibers of cellulose acetate/metal organic framework-third generation PAMAM dendrimer for the removal of methylene blue from aqueous media
Source: Sci Rep. 2023 Mar 25;13:4924. doi: 10.1038/s41598-023-32097-3 (PMC10039946; doi:10.1038/s41598-023-32097-3)
Supplement: Supplementary file 2 — Supplementary Information 2. [file 41598_2023_32097_MOESM2_ESM.doc]

**Supplementary information 2**


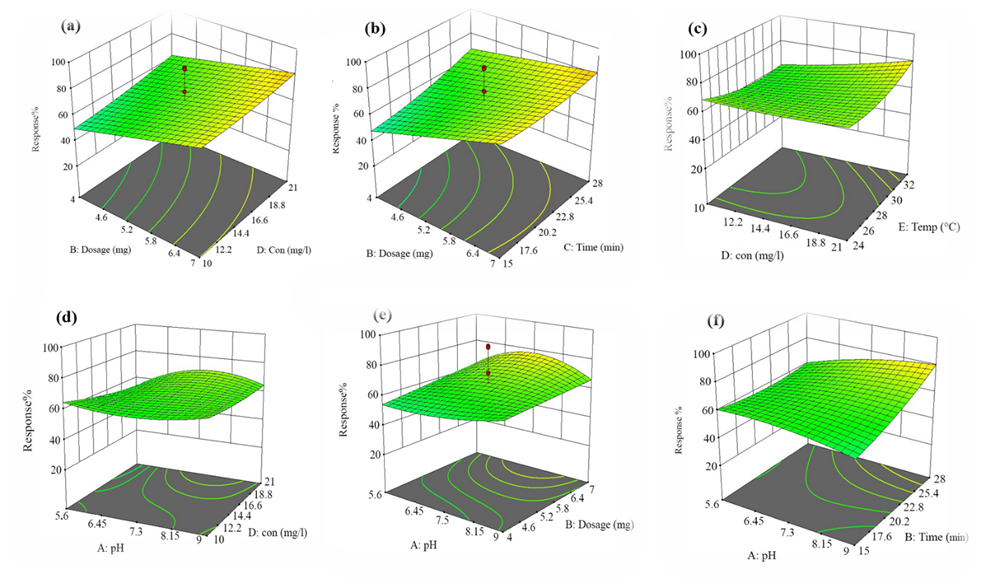

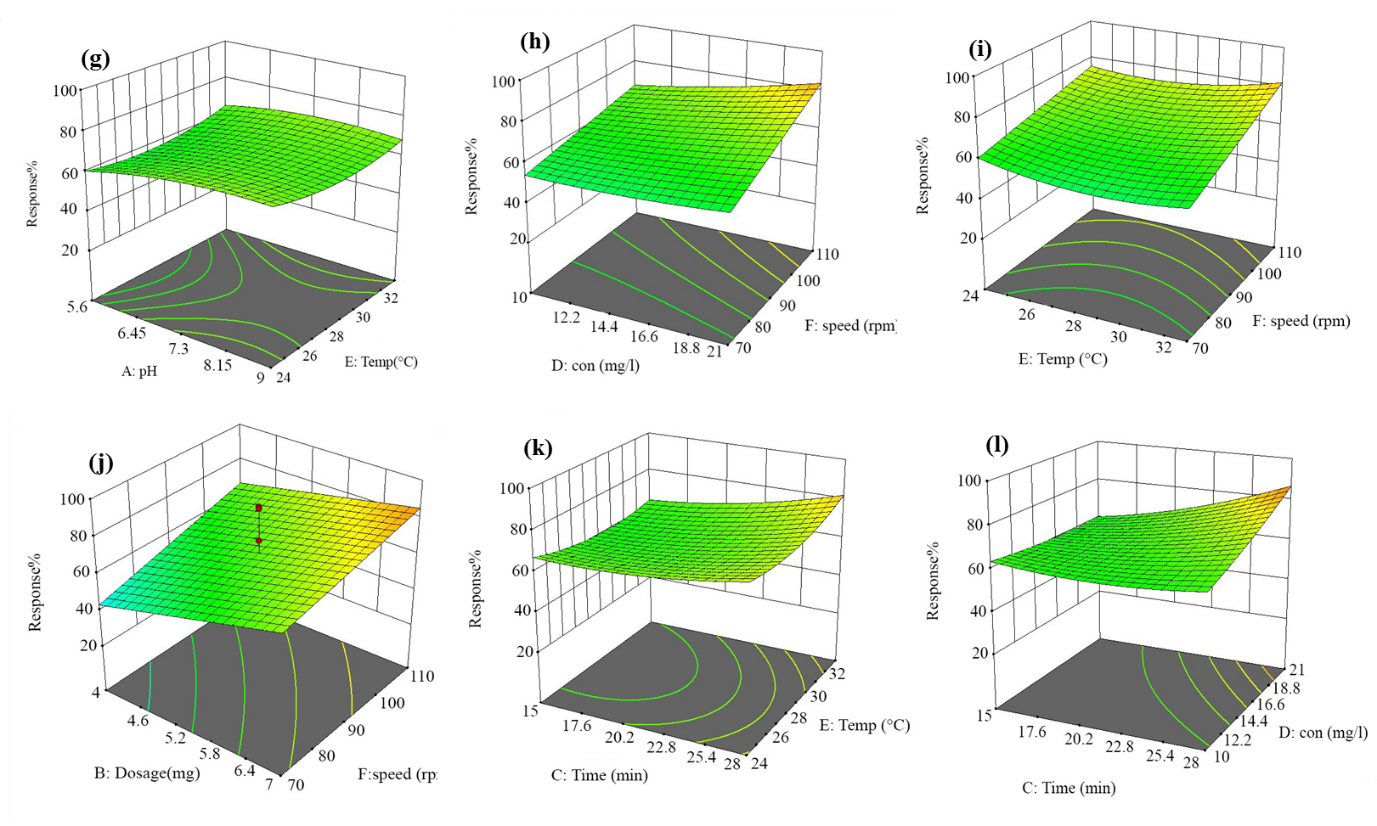

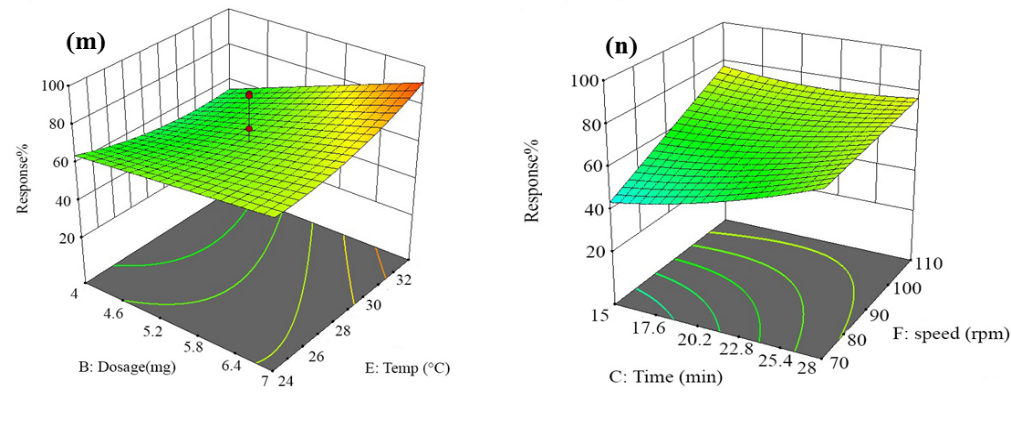


**Supplementary information 2:** 3D plots exhibiting the impact of various parameters on MB adsorption by the nanofiber adsorbent: (a) dose and concentration; (b) dose and time; (c) concentration and temperature; (d) pH and concentration; (e) pH and dose; (f) pH and time; (g) pH and temperature; (h) concentration and speed of stirring; (i) temperature and speed of stirring; (j) dose and speed of stirring; (k) time and temperature; (l) time and concentration; (m) dose and temperature; (n) time and speed of stirring.
